# Increased Expression of AQP 1 and AQP 5 in Rat Lungs Ventilated with Low Tidal Volume is Time Dependent

**DOI:** 10.1371/journal.pone.0114247

**Published:** 2014-12-09

**Authors:** Gustavo Fabregat, José García-de-la-Asunción, Benjamín Sarriá, Julio Cortijo, José De Andrés, Manuel Mata, Ernesto Pastor, Francisco Javier Belda

**Affiliations:** 1 Hospital General Universitario, Valencia, Spain; 2 Hospital Clínico Universitario, Valencia, Spain; 3 Department of Pharmacology, School of Medicine, University of Valencia, Valencia, Spain; 4 Research Foundation of Hospital General Universitario, Valencia, Spain; 5 School of Medicine, University of Valencia, Valencia, Spain, andAnaesthesia and Critical Care Department, Hospital General Universitario, Valencia, Spain; 6 Department of Physiology, School of Medicine, University of Valencia, Valencia, Spain; 7 School of Medicine, University of Valencia, Valencia, Spain, and Anaesthesia and Critical Care Department, Hospital Clínico Universitario, Valencia, Spain; 8 Instituto de Investigación Clínica (INCLIVA), Fundación de Investigación Hospital Clínico Universitario, Valencia, Spain; University of Valencia, Spain

## Abstract

**Background and Goals:**

Mechanical ventilation (MV) can induce or worsen pulmonary oedema. Aquaporins (AQPs) facilitate the selective and rapid bi-directional movement of water. Their role in the development and resolution of pulmonary oedema is controversial. Our objectives are to determine if prolonged MV causes lung oedema and changes in the expression of AQP 1 and AQP 5 in rats.

**Methods:**

25 male Wistar rats were subjected to MV with a tidal volume of 10 ml/kg, during 2 hours (n = 12) and 4 hours (n = 13). Degree of oedema was compared with a group of non-ventilated rats (n = 5). The expression of AQP 1 and AQP 5 were determined by western immunoblotting, measuring the amount of mRNA (previously amplified by RT-PCR) and immunohistochemical staining of AQPs 1 and 5 in lung samples from all groups.

**Results:**

Lung oedema and alveolar-capillary membrane permeability did not change during MV. AQP-5 steady state levels in the western blot were increased (p<0.01) at 2 h and 4 h of MV. But in AQP-1 expression these differences were not found. However, the amount of mRNA for AQP-1 was increased at 2 h and 4 h of MV; and for AQP 5 at 4 h of MV. These findings were corroborated by representative immunohistochemical lung samples.

**Conclusion:**

In lungs from rats ventilated with a low tidal volume the expression of AQP 5 increases gradually with MV duration, but does not cause pulmonary oedema or changes in lung permeability. AQPs may have a protective effect against the oedema induced by MV.

## Introduction

Mechanical ventilation (MV) has been used in critical care patients for decades. In spite of its life-saving potential, it has several shortcomings. A number of experimental studies have shown that mechanical ventilation may result in the appearance of inflammatory mediators in the lung [1] and subsequently in oedema. [2] Ventilator-Induced Lung Injury (VILI) causes macro and microscopic unspecific changes[3] similar to those found in patients with Acute Respiratory Distress Syndrome (ARDS). As it happens with ARDS, VILI is basically the result of important changes in the permeability of the alveolar-capillary membrane. [4] The potential of mechanical ventilation for triggering or worsening pulmonary damages has been shown in animal models where the application of non-physiological ventilatory parameters (mostly very high tidal volumes) aggravated the condition of animals with a previously injured lung [5], and even caused an injury in those without a previous pulmonary pathology. [2] The use of low tidal volumes has proved to be a better approach in ARDS patients, survival being improved in strategies based on its usage. [6]–[8] Interestingly, recent experimental and clinical work has demonstrated that MV with low tidal volume can induce similar pulmonary changes to those noticed for VILI [9]–[11] and that its appearance may be related to MV exposure time. [12]

Aquaporins are a family of small transmembrane proteins that help water to move fast, selectively and bi-directionally through lipid bi-layers. [13], [14] 13 different types have been identified in mammals, [15] from which the lung is known to express four: AQP-1, in the pulmonary capillary endothelium (especially alveolar), and the visceral pleura; AQP-3, in the tracheal epithelium; AQP-4, in the tracheal and bronchial epithelium; and AQP-5, on type I pneumocyte cells of the alveoli, on the membrane adjoining to the alveolar lumen. [16] Their role in the development and resolution of pulmonary oedema gives rise to controversy, although it does seem to play a part in VILI. [18]

This research aimed to verify if MV with low or moderately high tidal volumes (10 ml/Kg) sustained over time results in lung injury, subsequently altering pulmonary water content and microvascular permeability, as observed in VILI, and to objectivize what happens with AQP 1 and 5 expression, both types mainly involved in the formation of lung oedema, under the same ventilation conditions.

## Material and Methods

### 1. Ethics statement

The project was carried out after approval from the Ethics Committee for Animal Experimentation and Wellbeing of the Research Foundation of Valencia's Hospital Clínico Universitario.

### 2. Animal model and monitoring

A total of 30 rats were anaesthetised by intraperitoneal injection of ketamine 80 mg/kg and xylazine 5 mg/kg. 5 rats (group C or controls) were sacrificed by intravenous injection of 100 mg/Kg thiopental. The rest of the animals (n = 25) were performed a surgical tracheostomy, using a teflon cannula (Surflo, 16G). Rats were randomly allocated into two groups. 12 rats were ventilated for 2 hours (group 2H) with a Harvard Rodent Ventilator, model 683 (Harvard Apparatus) with a tidal volume of 10 ml/kg and a respiratory rate of 90 breaths/minute. 13 rats were ventilated with exactly the same parameters for 4 hours (group 4H). The cervical vascular bundle was dissected, and the right internal jugular vein and the right carotid artery were catheterized to continuously monitor heart rate (HR) and mean arterial pressure (MAP). Peak inspiratory pressure and respiratory system compliance were continuously recorded.

Anaesthesia was maintained by continuous intravenous infusion of ketamine and cisatracurium using dosis of 100 mcg/Kg/min and 2–3 mcg/Kg/min, respectively (20 ml of ketamine 5%, 10 ml of cisatracurium 0,2% and 20 ml of saline solution 0,9% at a rate of approximately 0,1 ml/h in the internal jugular vein). Anesthesia was supplemented, in cases in which it was necessary, by administration of an intravenous bolus of 0.1 ml of the mixture. Gasometric samples were taken in all animals in groups 2H and 4H at the beginning of MV and 30 minutes before the end of MV.

Rats were sacrificed by intravenous injection of sodium thiopental. The left lung was used for the determination of lung water content. The right lung of 6 rats from groups 2H and 4H was used for determining AQP 1 and AQP 5 expression. Lungs were either frozen in liquid nitrogen or paraffin-embedded for immunohistochemical sectioning and marking. 2 rats in group C and 4 rats from groups 2H and 4H were used to establish pulmonary macrovascular permeability.

### 3. Measuring lung oedema

Lungs were dried with filter paper and placed on a Petri dish with known weight to obtain lung wet weight (LWW). They were then placed in a drying chamber at 80°C for 96 hours and their dry weight (LDW) was determined.

Two indicators of the amount of oedema were obtained: Lung WW/DW ratio and the proportion of pulmonary water, expressed in percentages (%_ water_). The latter parameter was estimated using this formula: % _water_  =  (LWW - LDW)/LWW * 100

### 4. Measuring microvascular permeability

Microvascular permeability was quantified using Evans Blue Dye. 0.5 ml Evans Blue was injected intravenously (30 mg/Kg) 30 minutes before sacrificing the animal. Rats were sacrificed by exsanguination from the carotid artery, but saline was simultaneously infused via the jugular vein in the same amount as that of the blood extracted.

After death, the right lung was separated and immersed in formamide (5 ml) and homogenised for 2 min. The resulting suspension was incubated at 37°C/18 h and then centrifuged at 5000xg/30 minutes, and the supernatant was measured. Concentration of Evans Blue in the supernatant was spectrophotometrically determined.

### 5. Study of aquaporin expression

#### 5.1 Western blot

Proteins were extracted from previously frozen lungs. A Compartmental Protein Extraction Kit (Chemicon International, Temecula CA) was used. 200–400 mg tissue was homogenised in cold buffer C (1 ml/g tissue) and Ultra Turrax (KA, Staufen, Germany). Two protein fractions were obtained for each sample: cytoplasm and membrane, and they were quantified. Proteins in each fraction (100 mg) were separately run on a Tris-HCl/SDS gel, 8% acrylamide, and they were transferred onto a nitrocellulose membrane (Hybond-ECL, Amersham). After washing the membrane with distilled water, it was blocked with a PBS/Tween solution, 0.2%, with 5% skimmed milk. It was then incubated with the primary antibody during 2 hours at room temperature. The antibodies used were Anti-Rat AQP1 (Alpha Diagnostic, San Antonio, TX) and Anti-Rat AQP5 (Alpha Diagnostic, San Antonio, TX), both with a 2 mg/ml concentration. After several washes with PBS/Tween 0.2%, it was incubated with the secondary antibody Anti-Rabbit IgG (DAKO, Glostrup, Denmark) in 1∶2000 dilution. β-actin expression was detected as an internal control, and relative protein content was analysed using the enhanced chemoluminiscence method.

#### 5.2 Real-time polymerase chain reaction with reverse transcriptase

Lungs were cut with a microtome, three sections being obtained for each sample for total RNA extraction with TRIZOL (Reagent InvitrogenTM Life Technologies) as in the phenol extraction method described by Chomczynsky. [19] Microsections were added 1 ml TRIZOL and homogenized (Polytron PT 1200, Kinematica AG) and centrifuged at 10.000 rpm/10 min at 4°C. The supernatant was removed and RNA was precipitated by adding 0.1 volumes of sodium acetate 3 M, 2.5 volumes cold ethanol and 0.5 µl glycogen (20 mg/ml). RNA was centrifuged again, air-dried and resuspended in 20 µl Tris/EDTA buffer. RNA was reversely transcribed to cDNA with Superscript II (Invitrogen), by incubation with reverse transcriptase at 50°C for 30 min, followed by amplification with custom primers (Invitrogen), summarised in Table 1. 35 amplification cycles were completed, with denaturalization at 95°C (30 sec), hybridization (30 sec) (temperatures on Table 1) and extension at 72°C (1 min). Following amplification, RT-PCR products were separated in agarose gels at 1% and bands were viewed by ethidium bromide staining, and quantified by band density scanning using Scion Image (Beta 4.02, Scion Corporation). Results were expressed in relation to the level of β-actin mRNA in the same RNA samples.

**Table 1:** RT-PCR primer sequences and temperature conditions.

Gene	Primer (5′-3′)	Primer, counterclockwise (5′–3)	T (°C)
AQP1	TCTGGAGGCTGTGGTGGCT	AAGTGAGTTCTCGAGCAGGGA	60
AQP5	TGGGTCTTCTGGGTAGGGCCTATTGT	GCCGGCTTTGGCACTTGAGATACT	50
β-Actin	ATCATGTTTGAGACCTTCAACA	CATCTCTTGCTCGAAGTCCA	56

#### 5.3 Immunohistochemical study

Sections (4 µm thickness) from the paraffin-embedded lungs were obtained with the microtome. After the sections were dewaxed and hydrated, autoclave pretreatment (10 min, 121°C) for AQP1 and AQP5 antigen retrieval was performed and the sections were incubated in 1% H2O2 for 30 min at room temperature to block endogenous peroxidase activity. After being washed in PBS, the sections were then preincubated with goat serum albumin for 30 min at 37°C, and subsequently incubated with the primary antibodies against AQP1 (1∶500) and AQP5 (1∶300) for 18 h at 4°C. Then, the sections were washed with PBS and stained with Biotin-labelled goat anti-rabbit IgG for 30 min at 37°C. Intervening washes in PBS again were followed by incubation with Horseradish enzyme labelled streptavidin working solution for 30 min at 37°C. The sections were washed in PBS before application of diaminobenzidine (DAB), then were mounted under coverslip and analyzed under light microscope.

### 6. Statistical analysis

Results were expressed as mean ± standard deviation (SD). To compare results between groups, the non-parametric Kruskall-Wallis and Mann-Whitney tests were used. For the analysis of data within each individual group, the Wilconxon test was applied. Regression analyses for the amount of aquaporins and mRNA and determination coefficients (R^2^) were performed. Values of p<0.05 were assumed to be statistically significant in all cases.

## Results

### 1. Pulmonary oedema

**Figure 1:**
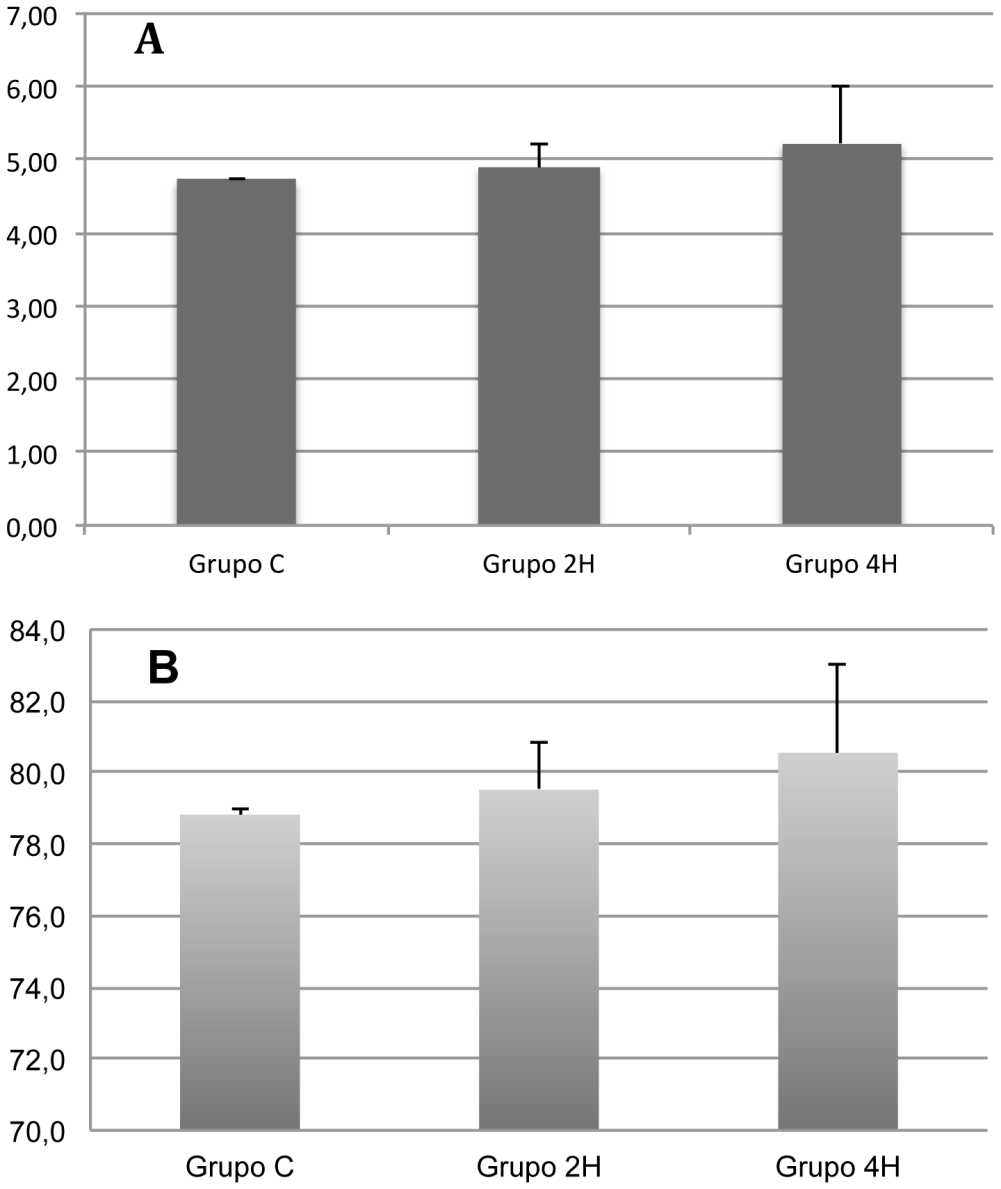
Pulmonary water content charts. **A.** The bar chart shows the results of lung wet weight/dry weight ratio (WW/DW). **B.** Graphic representation of pulmonary water content (%_water_). Error bars represent standard deviation. Group C  =  Control rats; Group 2H  =  Rats ventilated with 10 ml/Kg tidal volume for 2 hours; Group 4H  =  Rats ventilated with 10 ml/Kg tidal volume for 4 hours.

No significant differences were found between the three groups for the wet weight-dry weight ratio (group C: 4.72±0.04 vs. group 2H: 4.90±0.33 vs. group 4H: 5.23±0.79) or the percentage of pulmonary water (group C: 78.82±0.16 vs. group 2H: 79.52±1.31 vs. group 4H: 80.55±2.50), though an increasing trend was noticed for both parameters (Fig. 1).

### 2. Ventilatory mechanics and hemodynamic parameters

**Figure 2:**
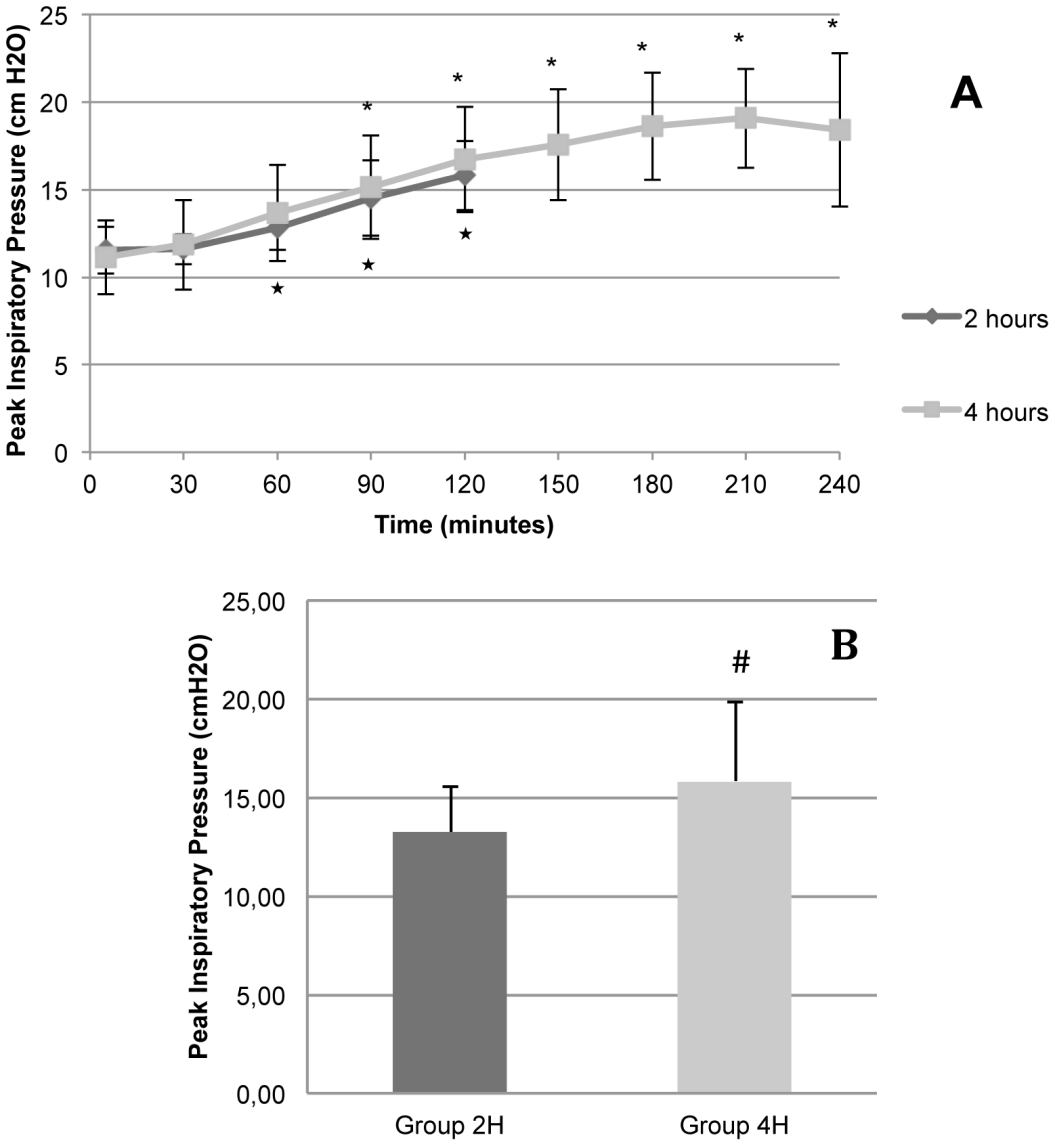
Peak inspiratory pressure. The figure shows the results of animals in Groups 2H (ventilated for 2 hours) and 4H (ventilated for 4 hours). **A.** Evolution of peak inspiratory pressure in relation to time. **B.** Graphic representation of mean peak inspiratory pressure in Groups 2H and 4H. Error bars represent standard deviation. Group 2H  =  Rats ventilated with 10 ml/Kg tidal volume for 2 hours; Group 4H  =  Rats ventilated with 10 ml/Kg tidal volume for 4 hours. ^*^ p<0.05 in relation to baseline of Group 2H. * p<0.05 in relation to baseline of Group 4H. # p<0.05 in relation to Group 2H.

Peak inspiratory pressure (PIP) progressively rose in both groups (group 2H and group 4H), higher values being found 90 minutes after the start of the experiment in group 2H and at minute 60 in group 4H (Fig. 2). No differences were found in the cut-off points of the two groups.

**Figure 3:**
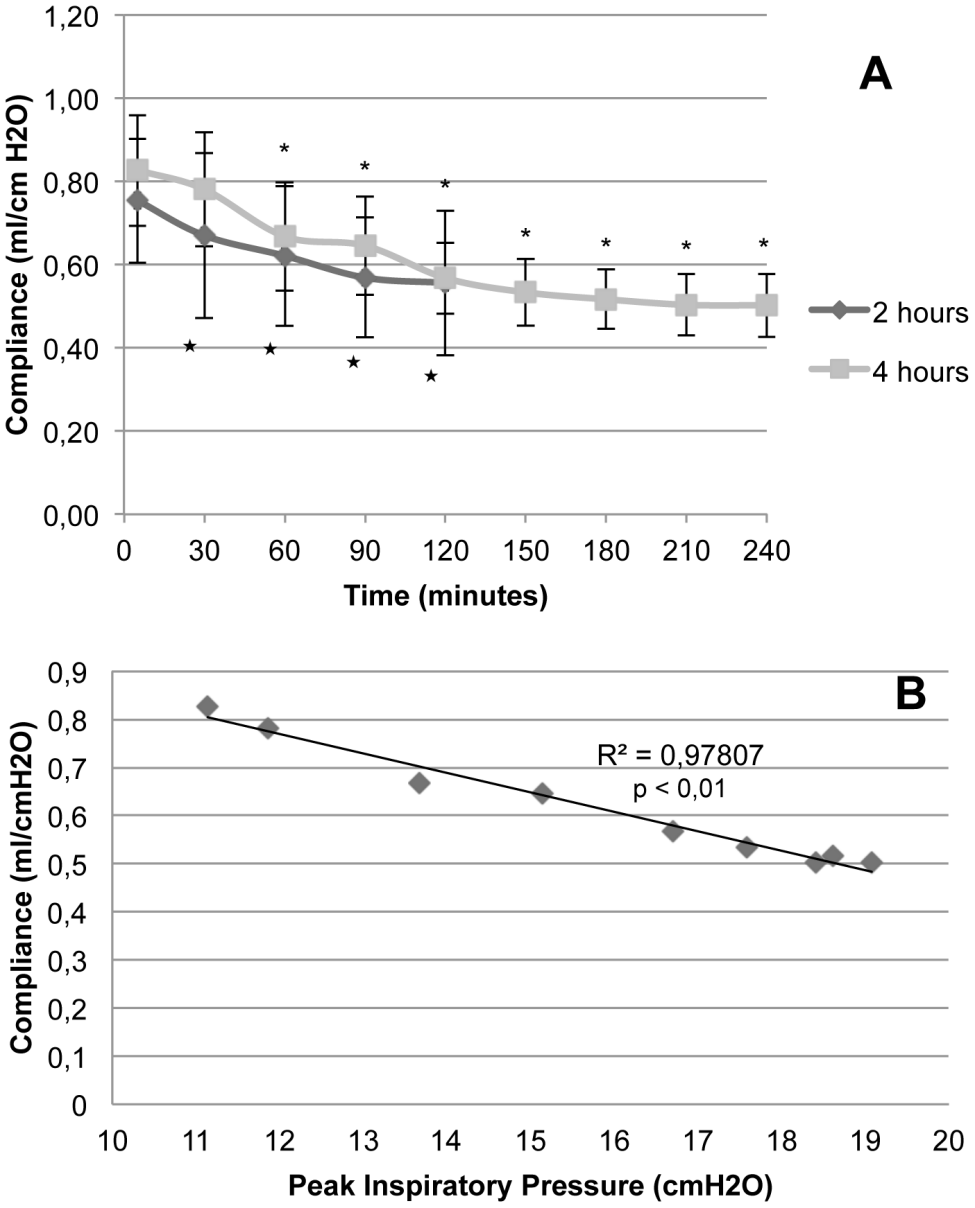
Pulmonary compliance. **A.** Evolution of animals in Groups 2H (ventilated for 2 hours) and 4H (ventilated for 4 hours) in relation to time. Error bars represent standard deviation. **B.** Dispersion chart and trend line for variation in pulmonary compliance in relation to peak inspiratory pressure in Group 4H animals (ventilated during 4 hours). *p<0.05 in relation to baseline of Group 2H. # p<0.05 in relation to baseline of Group 4H.

Pulmonary compliance was reduced gradually in both groups, with significance in respect of the initial value as from minute 30 for group 2H and minute 60 in group 4H (Fig. 3A). No differences were found between the groups in none of the cut-off points. This decrease in compliance correlated with the peak pressure rise, with an R^2^ value of 0.98, p<0.01 (Fig. 3B).

**Figure 4:**
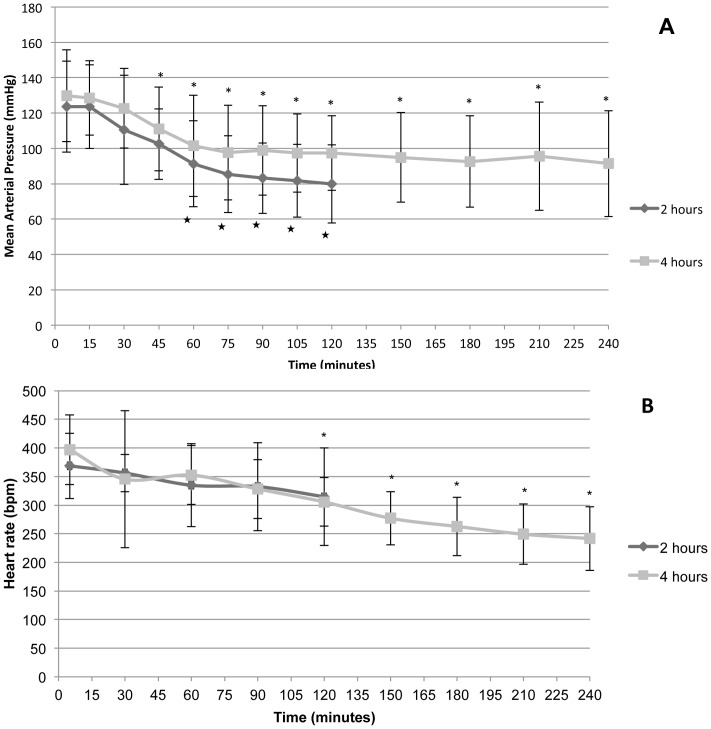
Evolution of hemodynamic parameters. **A.** Mean arterial pressure (MAP) and **B**. heart rate (HR) in Groups 2H (ventilated for 2 hours) and 4H (ventilated for 4 hours). Error bars represent standard deviation. *p<0.05 in relation to baseline of Group 2H. * p<0.05 in relation to baseline of Group 4H.

Rats in both groups were hemodynamically stable. No differences were found in mean arterial pressure (group 2H: 97.95 mmHg ±27.75 vs. group 4H: 104.53 mmHg ±27.54), and the same applies to average heart rate values (group 2H: 341.29 bpm ±66.73 vs. group 4H: 306.52 bpm ±78.11). Mean arterial pressure (MAP) dropped in group 2H progressively compared to the baseline as from minute 60, but this also happened in group 4H as from minute 45. Heart rate also decreased after 120 minutes (Fig. 4).

### 3. Gasometric parameters

Gasometric results for groups 2H and 4H are summarised in Table 2. No differences were found for pH, pCO_2_, ABE and lactate values within the study groups, and differences between the two groups were not found either. But a tendency towards mixed acidosis in relation to duration of MV was observed. Oxygenation presented a tendency to pO_2_ and pO_2_/FiO_2_ ratio reduction two hours after MV in group 2H, which was slightly more marked in group 4H after 4 hours.

**Table 2:** Results of arterial blood gas tests in Group 2H (ventilated for 2 hours, 10 ml/Kg) and Group 4H (ventilated for 4 hours, 10 ml/Kg).

	Group 2H (2 hours)		Group 4H (4 hours)	
	Baseline	2 hours	Baseline	4 hours
**pH**	7,29±0,05	7,21±0,07	7,30±0,05	7,23±0,07
**pCO2**	47,96±3,56	56,87±18,04	47,33±8,51	52,84±7,73
**ABE**	−3,96±2,02	−6,70±1,61*	−3,70±2,72	−6,26±4,20
**Lac**	2,08±0,62	1,99±0,66	1,79±0,74	1,91±1,24
**pO2**	93,30±17,72	91,56±16,56	97,28±15,76	83,03±24,27
**pO2/FiO2**	444,38±84,62	436,86±79,17	462,78±74,61	395,22±115,30

Values expressed as mean ± standard deviation.

* p <0.05 in relation to baseline.

### 4. Microvascular permeability

A significant increase in microvascular permeability was not found. Evans Blue absorbance on lung tissue was as follows: 16.08 ng/mg ±2.45 in group C; 25.96 ng/mg ±9.90 in group 2H and 20.39 ng/mg ±2.20 in group 4H.

### 5. Expression of aquaporins 1 and 5

**Figure 5:**
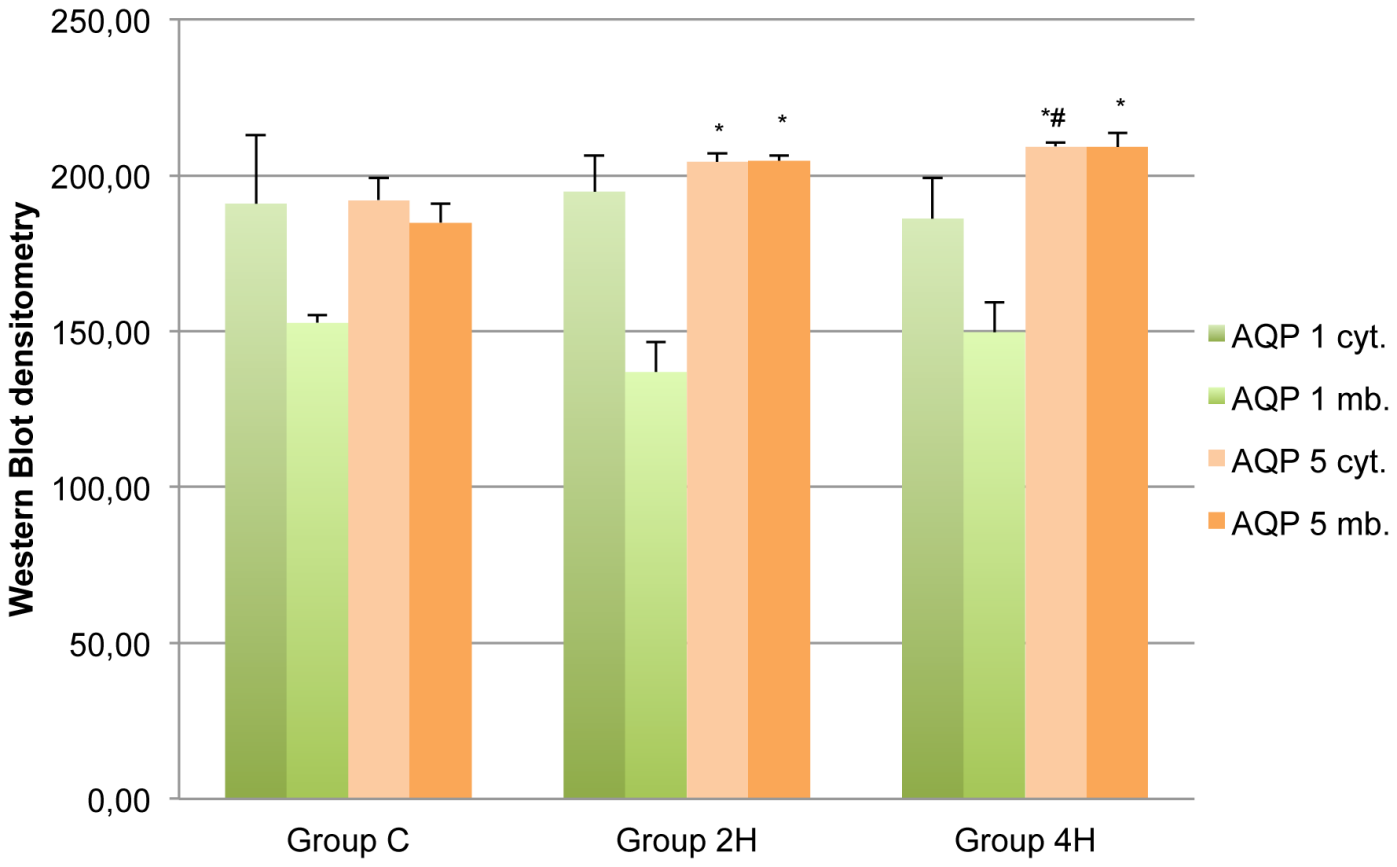
Western Blot densitometry values. Mean values. Error bars represent standard deviation. AQP 1 cyt.  =  Aquaporin 1, cytosolic; AQP 1 mb.  =  Aquaporin 1, membrane; AQP 5 cyt.  =  Aquaporin 5, cytosolic; AQP 5 mb.  =  Aquaporin 5, membrane. * p <0.05 in relation to value of Group C. # p <0.05 in relation to value of Group 2H.

**Figure 6:**
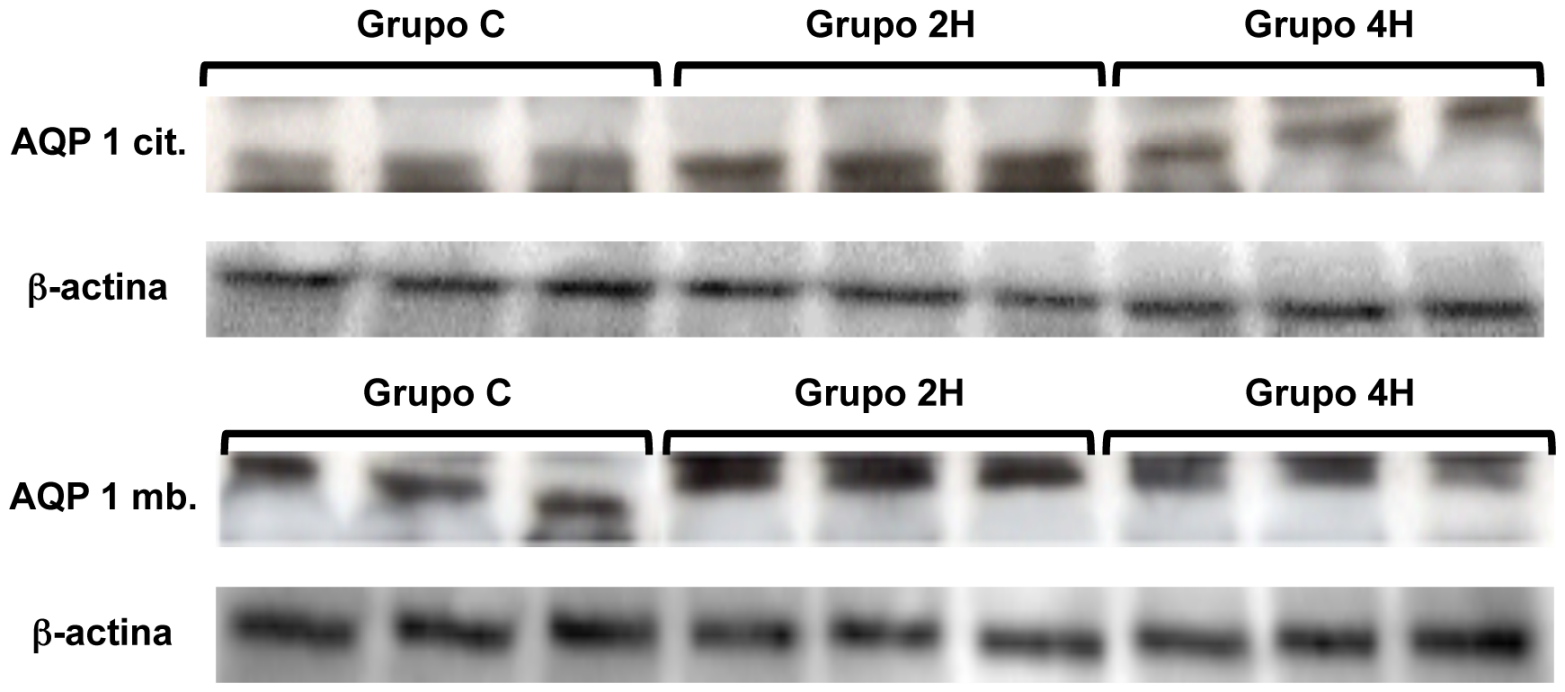
Western blot, AQP 1, cytosolic and membrane.

AQP 1 steady state levels measured by Western blot in membrane and cytoplasm did not show statistically significant differences in relation to duration of MV (Figs. 5 and 6).

**Figure 7:**
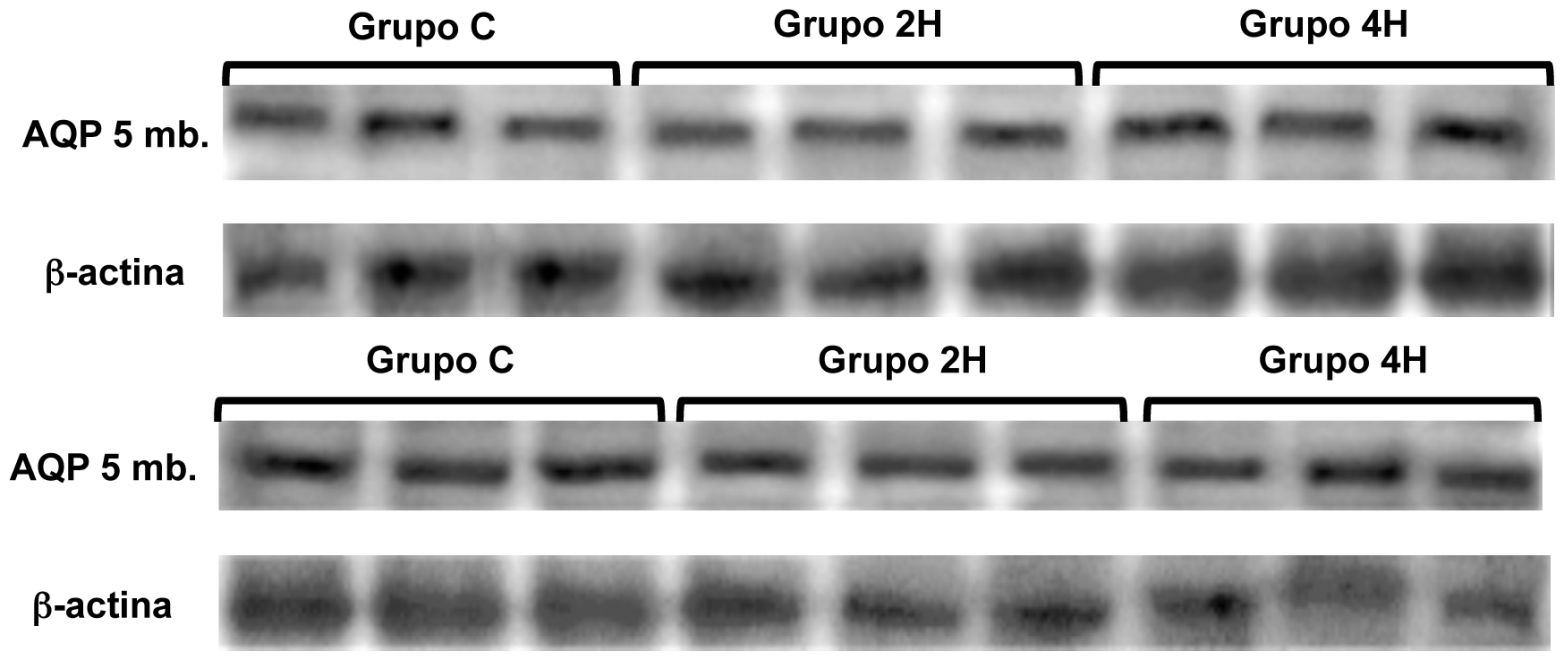
Western blot, AQP 5, cytosolic and membrane.

**Figure 8:**
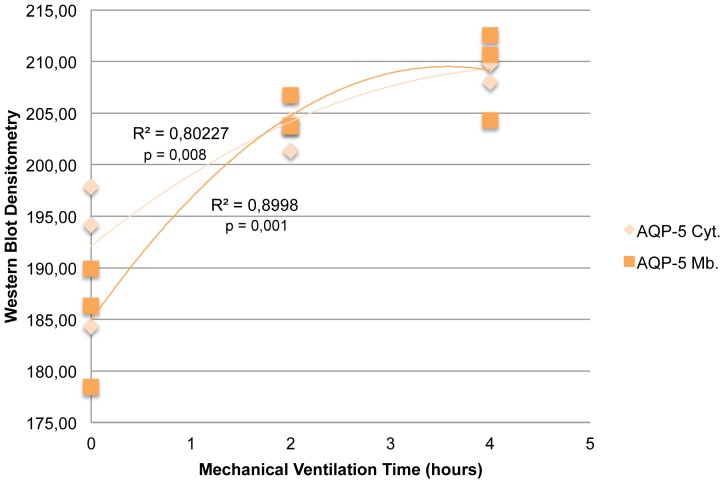
Dispersion chart and regression lines for Western Blot densitometries, AQP 5, cytosolic and membrane, in relation to time. Values correspond to dispersion coefficients R^2^. AQP 5 cyt.  =  Aquaporin 5, cytosolic; AQP 5 mb.  =  Aquaporin 5, membrane.

AQP-5 steady state levels in cytoplasm and membranes was significantly greater in both groups (2H and 4H) vs. the controls. Besides, AQP-5 expression on cytoplasm and membranes was greater in group 4H than in group 2H (p = 0.027 and p = 0.039, respectively) (Figs. 5 and 7). To better characterize these differences in the expression of AQP 5, a regression analysis of both variables was conducted, which provided a coefficient of determination R^2^ = 0.80 (p = 0.008) and R^2^ = 0.90 (p = 0.001) (Fig. 8).

**Figure 9:**
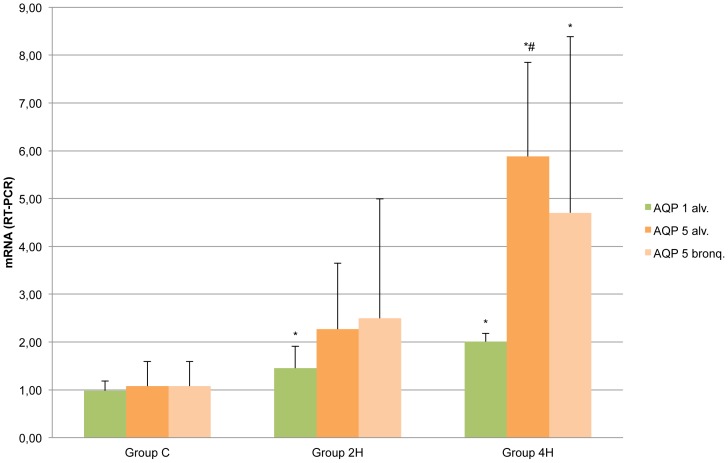
Representation of mRNA measured by RT-PCR. Error bars represent standard deviation. *p<0.05 in relation to value of Group C. # p<0.05 in relation to value of Group 2H.

RT-PCR results show a significant increase in the amount of mRNA for AQP-1 in groups 2H and 4H compared to group C. For AQP 5, an increase was found in the amount of mRNA in group 4H compared to group C (Fig. 9). The regression analysis of the amount of mRNA showed very significant determination coefficients for alveolar AQP 5 and AQP 1 in relation to duration of MV (R^2^ AQP-5 = 0.71 (p<0.001) and R^2^ AQP-1 = 0.69 (p<0.001).

**Figure 10:**
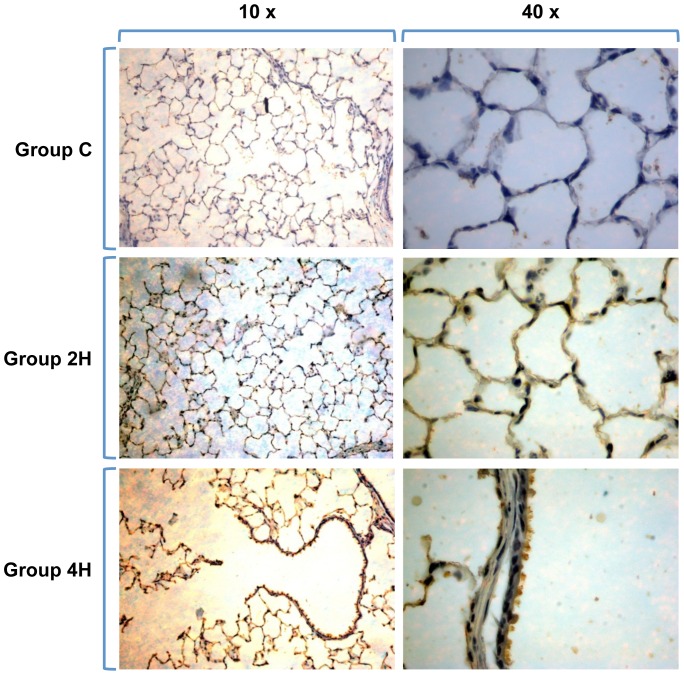
Inmunohistochemistry of AQP 5. The dye stakes the alveolar network and the surface of type 1 pneumocytes perfectly. Staining is more intense with longer MV exposure times (Groups 2H and 4H).

**Figure 11:**
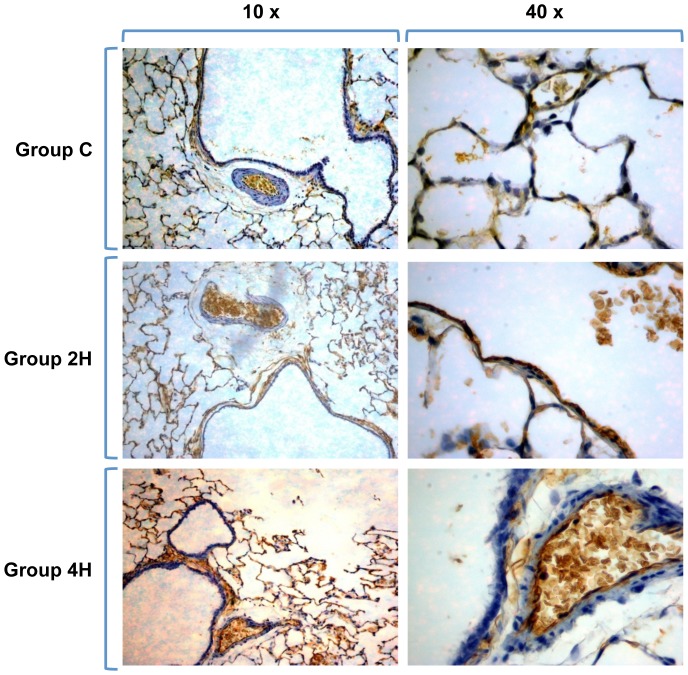
Immunohistochemistry of AQP 1. The dye demarks the microvascular network of capillaries around the alveoli, on the endothelium, of which AQPs-1 are preferentially expressed, as well as on the erythrocytes. No staining can be seen on type 1 pneumocytes.

Immunohistochemical lung preparations of AQP-5 show the membranes of type 1 pneumocytes delimiting the alveolar network. The intensity of the staining increases with duration of MV (Fig. 10). AQP 1 samples show the network of the alveolar capillaries, as AQP-1 is found in endothelial cells and red blood cells but not in the pneumocytes covering the alveolus. In this case, image analysis does not show an increase in dye intensity in relation to MV time (Fig. 11).

## Discussion

MV with high tidal volumes or without positive end-expiratory pressure (PEEP) may lead to the appearance of inflammatory mediators in the lung via mechanotransduction. [1], [20], [21] MV with tidal volumes over 12 ml/kg is associated with a bad prognosis, while "lung-protective ventilation" with low tidal volumes (under 10 ml/kg) and PEEP optimization reduces ventilation-induced injuries. [7]

However, ventilation with low tidal volumes may result in the appearance of an inflammatory response pattern in the lung. In rats ventilated with low pressures (12 cmH_2_O) and 4 cmH_2_O PEEP during 4 hours, increased activity of myeloperoxidase and macrophage inflammatory protein-2 and interleukin-6 has been reported. [9] Similarly, mild pro-inflammatory changes have been found in tracheal aspirates and blood of children with healthy lungs ventilated for 2 hours for heart surgery. [22] Likewise, 6 ml/Kg tidal volume MV in Wistar rats has been reported to induce a proinflammatory and profibrogenic response in the lung. [23] And in mechanically ventilated mice at 7.5 ml/Kg for 5 hours, rising levels of IL-6, TNF-α and lung water were found. [11] Conversely, some trials have demonstrated MV with tidal volumes of 10 ml/kg during 6 hours not to cause an increase in cytokine expression. [24] Similarly, no increases in cytokines or mediators were found in previously healthy patients undergoing MV after one hour of exposure, even with tidal volumes of 15 ml/Kg. [25]

Based on our results, we fail to confirm that MV with scarcely injurious parameters during 4 hours causes acute lung damage or changes in pulmonary permeability with an increase in lung water. A mild trend was observed in our experiments, however. Although levels were not measured for any inflammatory mediator, based on previous studies, inflammatory mediators are likely to be increased by MV even with low tidal volumes.

General anaesthesia and the supine position are known to cause pulmonary atelectasis with predominance in the dependent region. [26] These give the lung a heterogeneous appearance in which atelectasis areas co-exist with aired and even overdistended areas and their corresponding transition zones. [4] Besides, the use of low tidal volumes without PEEP can result in the appearance and maintenance of atelectasis in patients under general anaesthesia and muscular relaxation. [11] Regular recruitment with frequent deep insufflations during low tidal volume MV has been reported to improve oxygenation without signs of lung injury in mice mechanically ventilated for hours. [27] Therefore, this would explain the worsening observed in the oxygenation of our animals as exposure to MV with low tidal volumes and without PEEP increased.

Our animals may have developed atelectasis in dependent areas, causing different degrees of atelectrauma that would explain the degradation in oxygenation seen in the experiment (*see* Table 2), although it was not statistically significant, as well as the fall in pulmonary compliance and the rise in peak inspiratory pressure (*see* Figs. 2 and 3A). The latter parameters correlated linearly with a coefficient of determination R^2^ of 0.98 in group 4H (animals ventilated for 4 hours) (Fig. 3B).

Although acid-base parameters are well-known reliable indicators of animal wellbeing, they have only been partially evaluated in murine mechanical ventilation models. [9], [28], [24], [20] In our model, ventilated animals in groups 2H and 4H showed a trend towards mixed acidosis but without significance. As a result of the formation of atelectasis and higher pressures, ventilation might have been less effective, which could account for the rise in pCO_2_ levels, although not significantly. The metabolic component of acidosis can have several causes. Metabolic acidosis in mice can be induced by saline administration, [29], [11] which in our animals was used for maintenance at very low levels. Yet, metabolic acidosis caused by some hemodynamic failure cannot be totally excluded. This is possibly related to poor compensation for losses rather than potential deficits in venous return and secondary low cardiac output, as a result of the thoracic pressure reversal observed in positive pressure MV. [30] Similarly, heart rate data showed a significant reduction over time from minute 120 of the experiment, although always within the physiological range of rats. [31]

To date, no research group has explored the expression of AQP 1 and AQP 5 jointly in mechanically ventilated rats with low tidal volumes. However, a study conducted in rats ventilated with very high tidal volumes (40 ml/Kg during 4 hours) showed a reduction in AQP 1 expression. The researchers suggested an inflammatory mediator secondary mechanism, as they managed to mitigate the reduction in AQP 1 by administering a cyclooxygenase 2 inhibitor. [32] In another trial, based on an animal model of ARDS induced by smoke inhalation, animals were subjected to MV, finding an increase in mRNA for AQP 1. [33] The shortcoming in these studies is the absence of a control group with non-ventilated animals to study AQP expression.

Our experiments show an increase in AQP 5 expression that became more significant as MV exposure was prolonged and, similarly, an increase in mRNA of AQP 5 which was also greater in group 4H compared to non-ventilated animals. These increases were not accompanied by significant variations in lung water content or microvascular permeability. Although an increase in AQP 1 in the lungs was not found, after 4 h of MV, our study did find a significant increase in mRNA. This is likely to be the previous step to AQP 1 synthesis. It is possibly due to the fact that a longer MV period is needed for an increase in AQP 1 on cytoplasm and membranes to be seen with the Western blot. Different factors have been shown to modulate the amount of AQPs, reducing them under a number of pathological conditions and leading to an increase in pulmonary water and oedema. [34]–[36] In addition, the fact that lung injury parameters improve when their expression is induced, assigns AQPs a protective mechanism in the occurrence and development of pulmonary oedema. [37]–[39] Although some studies with genetically modified animals debate the possible relevant role of AQPs in the reabsorption of alveolar water, they prove the importance of these channels in pulmonary permeability. [17], [14], [40] Significant changes in lung water or microvascular permeability were not objectivized in our animals. The explanation for this could be that AQPs, according to some authors, only work in situations of stress. [18] Further studies are needed to determine the true role of AQP1 and AQP 5 in MV.

To conclude, in our model prolonged MV and tidal volumes of 10 ml/Kg for 4 hours did not result in increased pulmonary water or changes in microvascular permeability, these being mechanisms involved in ventilation-induced lung injury. Our study found an increase in the protein expression of AQP 5 and its mRNA, correlated with exposure time and mechanical ventilation. Likewise, the amount of mRNA for AQP 1 also increased in correlation with MV time. Apparently, AQP 5 and AQP 1 can have a protective effect against MV-induced pulmonary oedema, but more studies are needed to clarify whether these proteins really play a relevant role in mechanically ventilated lungs under different conditions.
